# What do patients think about home-based testing for better asthma diagnosis? Insights from a qualitative study

**DOI:** 10.1136/bmjopen-2025-109347

**Published:** 2026-03-23

**Authors:** Binish Khatoon, Joanna Smith, Stephen Fowler, Angela Simpson, Clare Murray, Ran Wang

**Affiliations:** 1 Division of Immunology, Immunity to Infection and Respiratory Medicine (DIIIRM), School of Biological Sciences, Faculty of Biology, Medicine and Health, University of Manchester, Manchester, UK; 2 School for Health and Social Care, Sheffield Hallam University/Sheffield Children’s NHS Foundation Trust, Sheffield, UK; 3 Centre for Respiratory Medicine and Allergy, Institute of Inflammation and Repair, Manchester Academic Health Science Centre, University of Manchester, Manchester, UK; 4 Manchester University NHS Foundation Trust, Manchester, UK

**Keywords:** Asthma, Digital Technology, Telemedicine, Feasibility Studies, QUALITATIVE RESEARCH

## Abstract

**Abstract:**

**Background:**

Asthma is misdiagnosed in one-third of patients . Due to its variable nature, international guidelines recommend performing key diagnostic tests during symptomatic periods or in the morning to improve accuracy. Limited access to timely clinic appointments and community-based diagnostics makes this difficult. Handheld spirometry and fractional exhaled nitric oxide (FeNO) are feasible for home use, enabling timely and flexible testing.

**Objective:**

To explore patients’ views on performing spirometry and FeNO at home during the asthma diagnostic process.

**Design:**

A qualitative study using semistructured interviews. Data were analysed using the framework approach.

**Setting:**

This prospective observational study was conducted at a National Institute for Health and Care Research Clinical Research Facility, based within a large National Health Service Trust, as part of the Rapid-Access Diagnostics for Asthma (RADicA) study (ISRCTN11676160).

**Participants:**

A purposive sample of 15 symptomatic adult patients with general practitioner-suspected asthma who were referred for diagnostic evaluation of the condition; all patients were given home spirometry and FeNO devices during their diagnostic processes.

**Results:**

Three themes emerged from the analysis: ‘Perceived value of, and burdens of home testing’, ‘Views on device usability and acceptability’ and ‘Information and support needs’. Home testing was generally welcomed by patients as a way of improving their understanding of their condition and enabling an accurate diagnosis of their symptoms. Key barriers (eg, testing frequency, lack of privacy) and enablers to improve feasibility (eg, training and support) were also identified.

**Conclusion:**

This study provides valuable insights into the barriers and enablers of home-based diagnostic strategies for asthma. Findings can inform service design and implementation approaches to enhance the feasibility and effectiveness of home testing.

**Trial registration number:**

ISRCTN11676160.

**STRENGTHS AND LIMITATIONS OF THIS STUDY**

- A key strength of this study is that the patients participated in the interviews were representative of those at the diagnostic stage.
- Patients who participated in the interviews openly shared their experiences in the absence of the main clinical research staff.
- The identified enablers and barriers to ambulatory testing using handheld devices for asthma diagnosis are transferable to other settings, including for disease monitoring and self-management.
- The study findings may not be representative of the experiences and views of all patients with suspected asthma due to the qualitative nature of the study design, but nonetheless, the current study included a diverse group of patients with broad baseline characteristics, providing useful insights into their views.

## Introduction

 Asthma is a prevalent and heterogeneous chronic airways disease,^1^ characterised by fluctuating respiratory symptoms and variable airflow obstruction; this is usually associated with airway inflammation.^2^ Misdiagnosis in asthma is common: overdiagnosis affects one in three individuals who are diagnosed with and receiving treatment for the condition,^3^,^5^ while the underdiagnosis rate is estimated to be between 20% and 73%.^5^

Asthma symptoms are frequently non-specific; therefore, clinical symptoms must be supported by objective testing during its diagnostic process. However, despite its cardinal feature of temporal variability in both symptoms and pathophysiology,^26^,^9^ the clinical diagnostic approach for asthma relies on a ‘one-off’ clinic-based testing strategies, with tests performed at a prescheduled clinic regardless of whether patients are experiencing their typical symptoms at the time.^2 10^ Therefore, it is not surprising that neither single tests nor combinations of tests, performed during a planned clinic in primary care settings, are sufficiently effective in ruling in or ruling out asthma.^11^,^13^ The Global Initiative for Asthma^2^ recognised this limitation and recommends the key diagnostic tests to be performed when symptomatic or in the morning when possible, but this is rarely feasible in routine practice. Peak expiratory flow (PEF) has historically been the only accessible domiciliary test for asthma diagnosis, enabling longitudinal measurements in patients’ usual environment and at times of symptoms.^2 3^ However, its diagnostic efficiency is poor, limiting its clinical utility.^11 14^

Therefore, improving asthma diagnosis requires innovative approaches. Fractional exhaled nitric oxide (FeNO), a single-breath biomarker for type-2 airway inflammation, and spirometry are key clinic-based asthma diagnostic tools. Advances in digital technology now enable these tests to be carried out at home using portable devices. The UK’s 10 Year Health Plan prioritises digital healthcare and identifies respiratory health as a key focus for the coming years.^15^ Therefore, the introduction of domiciliary spirometry and FeNO for home-based testing in asthma diagnosis aligns with this agenda and supports efforts to harness the wider digital transformation in healthcare. Although home spirometry and FeNO have shown feasibility in asthma self-management,^16^,^18^ few studies have explored their acceptability and perceived utility specifically in the diagnostic setting.

In this study, we explored patients’ views on the perceived potential benefits, barriers and enablers of home-based testing using handheld spirometry and FeNO during the asthma diagnostic process. This is a critical early step in understanding stakeholder perspectives and assessing their clinical and implementation potentials in this setting.

## Methods

### Study and design

A qualitative descriptive design was adopted, as qualitative methods were appropriate to explore and describe patients’ perspectives and experiences of home-based testing for asthma diagnosis. The study methods and results are reported according to the Consolidated Criteria for Reporting Qualitative Research guidelines.^19^

### Participant recruitment and clinical test procedures

The study is nested in the Rapid Access Diagnostics for Asthma study (RADicA, https://www.radica.org.uk).^20^ Symptomatic and untreated adults (>16 years) with general practitioner-suspected asthma were recruited from primary care and provided with home spirometry (MIR Spirobank Smart, Intermedical, UK) and FeNO (NOBreath, Bedfont, UK) devices. With permission, the mobile software application (‘app’) associated with the spirometers was downloaded, set up and installed on the participants’ smartphones in the RADicA clinic during their appointments. The MIR Spiro software is compliant with the international American Thoracic Society/European Respiratory Society guidelines^21^ and standards for spirometry. The app software provides a virtual assistant for users to achieve optimum spirometry blow technique. In-person training and written instructions were provided for both devices; verbal and written explanations about what FeNO and spirometry measure and simple guides were given to participants regarding what the results may mean (see online supplemental figure E1). Self-reported digital confidence was assessed at the time of device training by asking: ‘How confident do you generally feel about using digital products and mobile phone apps?’ with five ordinal response options of ‘confident’, ‘somewhat confident’, ‘neutral’, ‘somewhat not confident’ and ‘not confident’.

Participants were asked to perform FeNO and spirometry tests four times a day, for example, 4–5 hourly (±1 hour) when awake (eg, 0600–0800, 1100–1300, 1600–1800, 2100–2400 hour) and whenever symptomatic (before salbutamol use) for 1 week. For the 2nd week, participants were asked to use the spirometer two times per day (0600–0800 and 2100–2400 hour) and whenever symptomatic. This schedule was designed to mirror the established 2-week PEF protocol used in routine asthma diagnosis to assess diurnal variability. As diurnal PEF monitoring over 2 weeks remains a recognised diagnostic parameter,^2 10^ we sought to evaluate whether home spirometry and FeNO could feasibly capture similar longitudinal variability within a similar time frame. The four-times-daily schedule in the 1st week is to enable characterisation of diurnal patterns in test parameters and identification of potentially optimal time points for future refinement.

The home spirometry results and flow volume loops were obtained through direct emailing of app-generated PDF reports to the study team; the recorded data were also directly downloaded from participants’ app when they returned to the clinic following the monitoring period. Participants were asked to write down FeNO results on a diary book (online supplemental figure E1), along with symptoms experienced during home monitoring period. Participants were also asked to record in the diary when tests were completed and reasons for missed tests. The diary book also contained simple written instructions of how to operate the devices, helpline and short explanations of what FeNO and spirometry measures and a simple guide for how to interpret results. Reminders were sent via text messages or emails, with participant permission, on day 3 and 5, with a follow-up phone call on day 7. Remote telephone support was available throughout the monitoring period. Participants were asked to return the devices and diaries following the home monitoring period. A total of 51 patients completed the 2 week home testing study which assessed the adherence and feasibility of performing these tests at home.

### Qualitative interviews

Participants who took part in the home testing clinical study were invited to take part in a semistructured interview; participants’ information sheet for the qualitative interviews was provided at the time of recruitment to the home-testing clinical study (prior to the 2-week home testing period).

The interviews were undertaken between Aug 2023 and May 2024 at the RADicA study clinic office in Manchester University NHS Foundation Trust, Greater Manchester, UK. Interviews were undertaken in person or via telephone, according to participants’ preference, after the 2-week home-testing period but within a maximum of 3 weeks following completion of home testing. Rapport was built during the consent process and at the start of each interview, when the purpose of the study and this stage of the research were further explained.

The 1:1 interviews lasted between 30–45 min; all interviews were conducted by BK, an experienced qualitative researcher, who was independent from the RADicA clinical research team. The interviewer had no specific prior knowledge or background in asthma or related health topics. BK was not involved in recruiting patients to the clinical study or in training patients to use the home diagnostic devices. All interviews were conducted in the absence of the RADicA clinical research team, allowing open sharing of their experiences and views.

An interview topic guide was developed with the input from our public and participant involvement and engagement activities and the wider study team (table 1). The semistructured interview style was adopted to ensure the aims of the study were addressed and focused on patients’ recent experiences of using the domiciliary diagnostic devices, the perceived benefits and challenges of home testing and potential areas for future improvement. Prompts were used flexibly to explore how their experiences fitted into participants’ everyday lives, and to expand and clarify areas when needed. Field notes were used to document initial reactions, emotions and cues which were not possible to capture on the recorder. With participants’ permission, the interviews were recorded, transcribed anonymously and analysed.

**Table 1:** Interview topic guide

Topic	Prompts/questions
Introductions and background	
Introductions Opening question How have your ‘asthma’ symptoms been?	Welcome, check the purpose of this interview Obtain consent and to audio record the interview
Experience of using home diagnostic devices	
Thinking back to using asthma testing devices, can you describe how you found using them? Were they easy to use? If so, why? If not, why not? Did you manage to complete all the tests? What were your opinions of the written and verbal instructions/training offered?	Ask about each device separately: Spirometry FeNO Ask about the design of the devices, ease of use and any difficulties experienced Depending on answer: Can you explain what motivated you to ensure you completed all tests? or Can you outline the reasons you were unable to complete all measures? Did you access support?
Perceived benefit or what’s good about home diagnosis	
What do you think about using the home diagnostic devices? From your point of view, how important would home testing be if it was part of your usual care?	What is good about the home diagnosis? Do you feel it is an acceptable way to assess if people have asthma? Can you explain your answers? What would encourage you to undertake home testing?
Perceived drawbacks or what’s not good about home diagnosis	
Do you think there are any drawbacks to home diagnosis testing?	If home testing was part of your usual care, what would prevent you from undertaking the testing?
Future improvement	
If you suggest three things we could improve on, what would they be?	In an ideal situation, what do you think would help to ensure effective home testing?
Closing the interview	Thank the participant for their time, explain what will happen to the results and ascertain whether they would like feedback

FeNO, fractional exhaled nitric oxide.

Study recruitment finished when BK, in discussion with JS, the qualitative research adviser independent of the clinical research team, recognised that participants were not offering any additional information and that the data were of sufficient depth to meet the study aims.^22^

### Data analysis

The framework approach was used to analyse the interview data, following the principles outlined by Ritchie and Spencer.^23 24^ This approach was selected as it allows systematic comparison across cases while remaining grounded in participants’ accounts.^24^

1. Data management: Recorded interview data and field notes were transcribed verbatim and uploaded into the qualitative data software package NVivo 12. BK undertook the initial coding, assigning ‘labels’ to data extracts. An initial codebook was developed inductively from the data during this stage, consistent with framework analysis procedures. Codes were defined, refined and documented as coding progressed. The developing codebook was discussed iteratively with JS to ensure clarity, consistency and alignment with the study aims.
2. Descriptive accounts: Once all transcribed data were assigned to preliminary subthemes, the codebook and thematic structure were reviewed and refined. Subthemes were examined for patterns, similarities and differences across participants, in line with the charting and mapping stages of framework analysis. Associations between subthemes were identified and explored. At key stages during the iterative development of subthemes, findings were discussed with the wider research team. These discussions took place across multiple team meetings and were used to challenge interpretations, resolve disagreements and reach consensus on theme development.
3. Exploratory accounts: Final themes and subthemes were agreed at team meetings, resulting in a cohesive narrative that met the overall study aims. This stage involved moving beyond descriptive categorisation to interpretation, examining how participants’ accounts related to broader behavioural, experiential and contextual factors influencing engagement with home-based spirometry and FeNO testing. This ensured that the findings reflected participants’ perceived benefits, burdens and potential barriers and enablers associated with the use of home-based spirometry and FeNO for asthma diagnosis.

In line with good qualitative research practice, a reflexive journal was kept by BK to document decisions throughout the analysis. Initial coding was carried out primarily by BK, with regular sharing of codes and reflections with JS. Codes and reflections were then reviewed by the wider research team (RW and CM, respiratory specialists) to refine interpretations and develop the final themes. This ensured consistency and transparency while allowing for deep engagement with the data and consensus agreement on the final themes. This approach is consistent with qualitative quality standards outlined in the Standards for Reporting Qualitative Research.^25^

Concordance was supported through shared review of coded extracts, iterative refinement of the codebook and consensus-based agreement on final themes. An audit trail documenting coding decisions, theme development and team discussions was maintained.

### Patient and public involvement

A Patient Advisory Group (PAG) comprising five members with asthma or caring for someone with asthma. Members of PAG co-designed the patient-facing materials, provided feedback on interview topic guides (table 1), diary book, written device training instructions, participant information sheets and consent forms. One PAG member also participated in the trial steering committee meetings.

## Results

Of 26 consecutively eligible participants invited, 16 patients were interviewed, with 1 participant’s recording corrupted and therefore not usable; 2 did not attend the appointment and 8 declined without giving a reason. Data collected from 15 participants with varying levels of demographic background, digital confidence and differing adherence to home testing were included in the analysis (table 2). The three overarching themes that emerged from the analysis were, ‘Perceived values and burdens of home asthma testing’, ‘Views on device usability and acceptability’ and ‘Information and support needs’ (table 3).

**Table 2:** Participant characteristics

Participant characteristics	(n=15)
Gender male: female	7:8
Diagnoses	
Asthma (n)	11
Not asthma (n)	4
Age (years)	Range: 21–52
18–30	5
31–40	5
41–50	1
Above 50	4
Ethnicity	
White British	10
Black Caribbean	1
Asian	1
Pakistani	1
Chinese	1
African	1
Employment status	
Student	3
Retired	3
Employed	7
Not in paid work	2
Self-reported digital confidence*	
Confident	10
Somewhat confident	3
Neutral	1
Adherence rate to home testing	
Home FeNO	
>75% adherence	11
50–75% adherence	2
25–50% adherence	1
<25% adherence	1
Home spirometry†	
>75% adherence	6
50–75% adherence	3
25–50% adherence	3
<25% adherence	3

* One response missing.

† An attempt was defined as performing at least two blows per measurement*.*

FeNO, fractional exhaled nitric oxide.

**Table 3:** Themes and subthemes

Themes	Subthemes
Perceived values of, and burdens of home asthma testing	Motivators to perform home testing
	Opportunity for condition self-management
	Limitations of home testing
Views on device usability and acceptability	Acceptability and usability of FeNO device
	Acceptability and usability of spirometry device and app
	Suggestions for improvement
Information and support needs	Information provision and modes of delivery
	Building confidence and ongoing support

FeNO, fractional exhaled nitric oxide.

### Theme 1: Perceived values of, and burdens of home asthma testing

A narrative throughout participants’ accounts was their *motivation for undertaking home testing,* and the opportunity for *self-managing their condition*, along with descriptions of *the limitations of home testing*.

*Motivators to perform home testing:* a desire to better understand asthma and asthma-related symptoms was a common motivator to undertaking home testing. Some participants expanded further, explaining that improved understanding of their condition and the meaning of test results would help them to know how well their lungs were functioning and therefore help them manage their condition. For most participants (11 out of 15), there was recognition that home testing could contribute to the accurate diagnosing of asthma, and participating in the study could help future patients. Examples of participants’ motivators included:

I feel like healthcare has an emphasis on dealing with problems once they’ve happened, whereas for me I think I’m very keen to look at sort of, you know, these little diagnostic tests that one can do before you get ill to find out if things are worsening or if things are beginning etc. …….It’s a way of learning more by doing the tests so you can categorically say for people in future that they can, they’re asthmatic or not basically (*Participant 7)*

I think the most important thing was whether or not this would help the doctors give me a more accurate diagnosis from my home monitoring, (*Participant 14)*

*Opportunity for condition self-management* while linked to the above sub-theme more explicitly related to participants’ desires to be more proactive in self-managing their condition (7 out of 15). They perceived this could be facilitated by home testing, through sharing and discussing results with health professionals. Some participants highlighted that home testing would empower them to act on their symptoms. Examples include:

Understanding the condition and then seeing, well, does anything need to be done? Or do I need to do something differently about the management of the condition? (*Participant 2)*

*Limitations of home testing*: while sharing results could help to self-manage their condition, for some this responsibility was overwhelming and they perceived some tasks unnecessary or overly complicated (5 out of 15). For example:

It’s not just the instructions, I think it’s more of like when you’re doing it and passing the information over through the internet. … And I don’t think you’d be getting the right readings because you’re doing something else rather than just concentrating on doing that, you know what I mean? Then sending it off. But if somebody was, say either on the phone or you’re doing it like through now and again a home visit or something, but, which contradicts the situation of doing it from home and….’ (*Participant 9)*

Similarly, one participant perceived that while asthma was a long-term condition, it was non-life threatening and that symptoms were to be tolerated. Therefore, they questioned the value of home testing if symptoms were unlikely to improve:

Because this thing is when people have asthma their condition is not going to improve like dramatically overnight. Like it’s a long-term thing. So they kind of get numb with their condition. They don’t think its anything so life threatening. That’s why this kind of task which takes time, takes effort becomes a burden not something they see benefit anymore. So if its more life-threatening thing it’s definitely very, very important, like heart disease you know heart attack or cancer. With asthma it’s kind of minor people don’t really put such weight on it…, *(Participant 4)*

For one participant, a drawback of home testing was an increased visibility of having asthma/asthma symptoms, with a perception this could be stigmatising and potentially impact on their mental well-being. As a consequence, this may influence undertaking the tests outside the home if they could not be done in private, for example:

It’s the mental weight that comes with it. There’s kind of a stigma when for instance, so if you’re in the home, other people have probably got to be there whilst you’re doing your test and whatnot, which- It makes you feel like you’re being- Sticking out like a sore thumb. And if you happen to be out and about and you have to do it in public for instance, …. it’s going to be detrimental to them in the long run as well, so that’s the kind of element I’d look at. Other people’s opinions and whatnot. And then obviously they might not want other people to know what’s going on as well, so it’s a privacy thing as well. (*Participant 5)*

### Theme 2: Views of device usability and test acceptability 

Participants shared their views of the *usability and acceptability* of both devices/tests and the associated spirometer app, *along with suggestions for improvement*. For many participants (9 out of 15), both tests were, once they became familiar with the devices, relatively easy to perform, for example:

I found it easy. Once you’d done the initial few, many of them and obviously, it’s straight forward. (*Participant 3)*

However, there were differences in the usability of the devices and tests as outlined below.

*Acceptability of the FeNO:* participants reported that the FeNO was easier to perform compared with the spirometry, which typically related to the breathing technique required to undertake the test and obtaining a result with a single effort (14 out of 15). For example:

I found that (FeNO) far easier and straightforward. But obviously, you’ve still got to master the technique, and maybe the technique is easier too, because you’ve got to get that flow just right. (*Participant 6)*

*Acceptability and usability of spirometer device and app:* portability was the main benefit of the spirometer device. A frequently reported limitation of the spirometer device was the number of times participants had to use the device to get an accurate reading; concerns about obtaining incorrect readings caused anxiety and frustration for some participants (13 out of 15). For some participants, completing the tests accurately appeared burdensome, with reports of feeling tired and frustrated if there had been many attempts to complete to get an accurate reading. Examples of the challenges reported include:

Because of more and more unsuccessful attempts …I started to wonder is there something wrong with the device and I just get some help and they tried to clean it and maybe there’s saliva wet inside. Just feel very frustrated because I need to get it done I feel like it’s a responsibility and also like a task but I can’t get a successful attempt and I have to repeat and my mouth’s very dry. (*Participant 4)*

I was just gasping for air. I’d blow into it, and I’d just be trying to catch my breath back because it just took a lot of energy out of me blowing into it, because you have to obviously hold your breath, and then do a long breath, …. I found it very difficult. (*Participant 1)*

The spirometer device was linked to an app, which in general participants reported the functions were easy to use (13 out of 15):

The app was great, so easy to use. They showed me how to use it anyway. And it was just, that was all right. It’s dead easy to use. But like I’ve got an iPhone so it’s just quite simple to just send the results over. (*Participant 11*)

While some participants highlighted the app was self-explanatory and the instructions were simple and easy to follow, others struggled to remember how to use the app and had technical difficulties (6 out of 15). For example:

I found that a bit more difficult to use in terms of it was a lot more complex, like the process. Because obviously, you had to get that app up. And also, I found it hard….I was doing my results a week and a half into it, they wouldn’t accept any of the results I tried to submit….I was paranoid that the team would have thought that I wasn’t doing it properly, I wasn’t doing what I was meant to do. But they were aware that the app was down, so it wasn’t that bad. (*Participant 1)*

*Suggestions for improvement*: Participants suggested the spirometry system could be redesigned, allowing uploading results via the app which could be instantaneous to health professionals rather than downloading and attaching to an email (7 out of 15). In addition, icons on the spirometry app could be simplified, including a clear explanation of their meaning. For example:

Surely there’s a way in the app where you can have it link up like so it’s almost like on a cloud service where you guys can see the results straight away rather than having to send an attachment. That was absolutely fine but like I’m sure, I’m sure there are ways you can have something on a cloud of some sort like just online and it’s like updated. That would just you know save a bit of time doing it because that was twice a day wasn’t it that one? (*Participant 13)*

…The pictures on the screen, I think it looks like it’s very small. I think the biggest picture was about yea big, which- If anybody’s hard of sight, regardless of age again, not going to be the most friendly and especially if they’ve got any memory issues as well…. (*Participant 5)*

While the FeNO device used in the current study was not designed for home use, as the feasibility study primarily assessed whether participants could perform FeNO testing at home. Nevertheless, overall participants reported the FeNO device to be simple and easier to use compared with the spirometry device. Participants reported the size of the device and having to change the tubes made it difficult for use outside the home (12 out of 15), highlighting design features that could be prioritised in future home FeNO devices. For example:

The purple one (FeNO) was absolutely fine, I mean the only thing that was a bit of a hassle with swapping out the thing, remembering to swap out the tubes … it was just big (*Participant 13)*

### Theme 3: Information and support needs

Participants highlighted that information and support were vital to the success of home testing, which included the way *information is provided* and *building confidence* and *on-going support*.

*Information provision and modes of delivery:* participants perceived that the training sessions were supportive, unrushed, carried out in a friendly environment and information provided was sufficient to prepare them to complete the tests at home independently, for example (14 out of 15):

Actually, at the hospital, when they handed over both devices, they showed me how to do it. I came back. I started doing it like, you know- It was easy and even, you know, in the book, the manual, at the hospital, I didn’t even check the manual because, you know, it was very easy.… the lady at the hospital, she showed me how to do it, how to use it…I was like, “It’s easy to be downloaded,” like, you know and she showed me how to do the app. (*Participant 8)*

They were very good actually, very good. Yes, very clear, very patient. I didn’t feel rushed to any of the tests, you know, all the stuff that we did that day in that first appointment, or indeed second one. You know, it didn’t feel rushed or anything. It was fine. (*Participant 15)*

However, some participants were concerned about forgetting the instructions and would have liked additional detailed instruction leaflets (6 out of 15), for example, how long to persevere to achieve an acceptable spirometer test result. Some participants suggested scheduled phone/face-to-face meetings from the clinical team (4 out of 15). Examples include:

I was, say, 70, and I was blowing 110 times, if I didn’t know anything about it, I’d just think, oh my God, this is horrendous. So you end up doing more then, you see. And obviously the more you do, the worse you get, and it just has a knock-on effect then. But yes, just a bit of info, really, about the blowing I’d have thought, upfront before you take it home. (*Participant 7)*

It was the last bit. There was a lot to take in and trying to work it out and then you were left- Obviously, you’re going home and you’re doing it, on your own basically. So, if it was more simplified and it was written down on maybe an A5 saying, this is what you need to do, and you could read it when you’re at home, that would make it a lot - For my personal preference. (*Participant 3)*

*Building confidence and ongoing support:* participants described that the knowledge, approachability and support, particularly when seeking advice, of staff was important in developing their confidence and motivating them to undertake home testing (14 out of 15). Examples of support included:

It makes you feel more confident that you’re being more looked after. You know the aftercare is still there even though you’re at home. I think it’s important and it gives you the motivation to carry on doing the tests. (*Participant 3)*

I think it’s pretty self-explanatory, but you could call the team if you had questions, which I think was good. And they did check up on you once or twice. That’s when I told them my problems with the orange one, I didn’t get any accepted trials and they said that the app is sometimes really sensitive. (*Participant 14)*

## Discussion

We found that home-based diagnostic approach is acceptable to patients in the asthma diagnostic setting, but perceived benefit and burden varied among individuals. Our findings highlighted the key perceived values, barriers and enablers of adopting digital health in the asthma diagnostic process. These findings form the basis for future products and service design for asthma diagnosis. These insights are also transferable to other contexts, including the use of digital devices for home monitoring to support asthma self-management. In line with the National Health Service Long Term Plan,^15^ promoting community-based and patient-centred care, there is an increasing emphasis on shifting diagnostic services closer to home. Therefore, understanding patient perspectives on these evolving models of care is essential to ensure uptake and effectiveness.^26 27^

Consistent with previous studies of home monitoring in other chronic lung diseases,^28^,^30^ we have demonstrated that home-based testing strategy using digital health devices has the potential to empower patient to be health proactive and actively engage in self-management of their conditions,^28^ providing a sense of control of their diagnostic journey. Furthermore, the early involvement of patients in their diagnostic process may facilitate shared decision‐making, potentially leading to better clinician–patient partnership in disease management and improved patient satisfaction, thereby resulting in better asthma control and overall improved quality of life.^31^

We have previously demonstrated that a home-based digital healthcare system (including hand-held spirometer and FeNO devices) had the potential to revolutionise the self-management of asthma, but adherence to two times per day measurements fell within the 1st month of testing^16^; test burden was identified as a common barrier.^32^ Longer duration and increased frequency of measurements represent an increased burden and form a significant barrier to this testing strategy. Nevertheless, compared with long-term monitoring, home testing to establish diagnosis requires shorter term engagement (over 1–2 weeks) and is likely to carry a better adherence rate. Notably, some patients perceived asthma as low priority and non-life threatening; therefore, they questioned the value of diagnostic testing. This perception stands in contrast to the substantial disease burden to individuals and society,^1 33 34^ highlighting the need for patient-centred communication about asthma at the point of testing to enhance uptake and adherence; this may also support subsequent treatment adherence and better self-management when asthma is confirmed.

While many identified barriers are modifiable through careful design and planning (such as user-friendly devices and app designs, ease of data capture, automated data sharing between healthcare professionals and patients, adequate training and support), others may be more challenging. For example, the feeling of lack of privacy and stigma of performing breathing tests in front of others and the challenges in breathing technique, particularly for spirometry. While the frequency and duration of home measurements may be minimised through future studies in determining the optimal timing of measurements, because spirometry is an effort-dependent, forced expiratory technique, repeated blows are mandatory for quality control and cannot be avoided. These challenges may be addressed through AI-based virtual assistance and emerging models such as telecoaching at home.^35^

### Strengths and limitations

We included a group of patients with varying testing adherence, diagnostic outcomes, ages, employment status and with a third from ethnic minority backgrounds. However, interviewed participants were a subselected group, limiting representativeness to all study participants. Nonetheless, the diversity of baseline characteristics in our sample provides valuable insights into patient perspectives.

Although we sampled participants with differing levels of self-reported digital confidence, most were digitally literate, with approximately a quarter reporting lower confidence, but none reported having low digital confidence. Our findings, therefore, are less representative of digitally excluded individuals. Furthermore, all participants were English-speaking. Understanding barriers and enablers among those at risk of digital exclusion and non-English speaking individuals is essential to ensure equitable delivery of digital healthcare and should be a priority for future research.

Subgroup analysis was not possible due to small sample size. While PAG members were involved in study design and delivery, they were not involved in data interpretation, potentially limiting the depth of patient-informed insight in the analysis.

## Conclusion

The barriers and enablers to home-based asthma diagnosis using handheld spirometry and FeNO should be carefully considered and optimised during service implementation. The clinical utility and cost effectiveness should be evaluated as the next step.

## Supplementary material

10.1136/bmjopen-2025-109347 online supplemental file 1

## Data Availability

Data are available upon reasonable request.
