# Supplementary figures and images for: What do patients think about home-based testing for better asthma diagnosis? Insights from a qualitative study
Source: BMJ Open. 2026 Mar 23;16(3):e109347. doi: 10.1136/bmjopen-2025-109347 (PMC13034331; doi:10.1136/bmjopen-2025-109347)

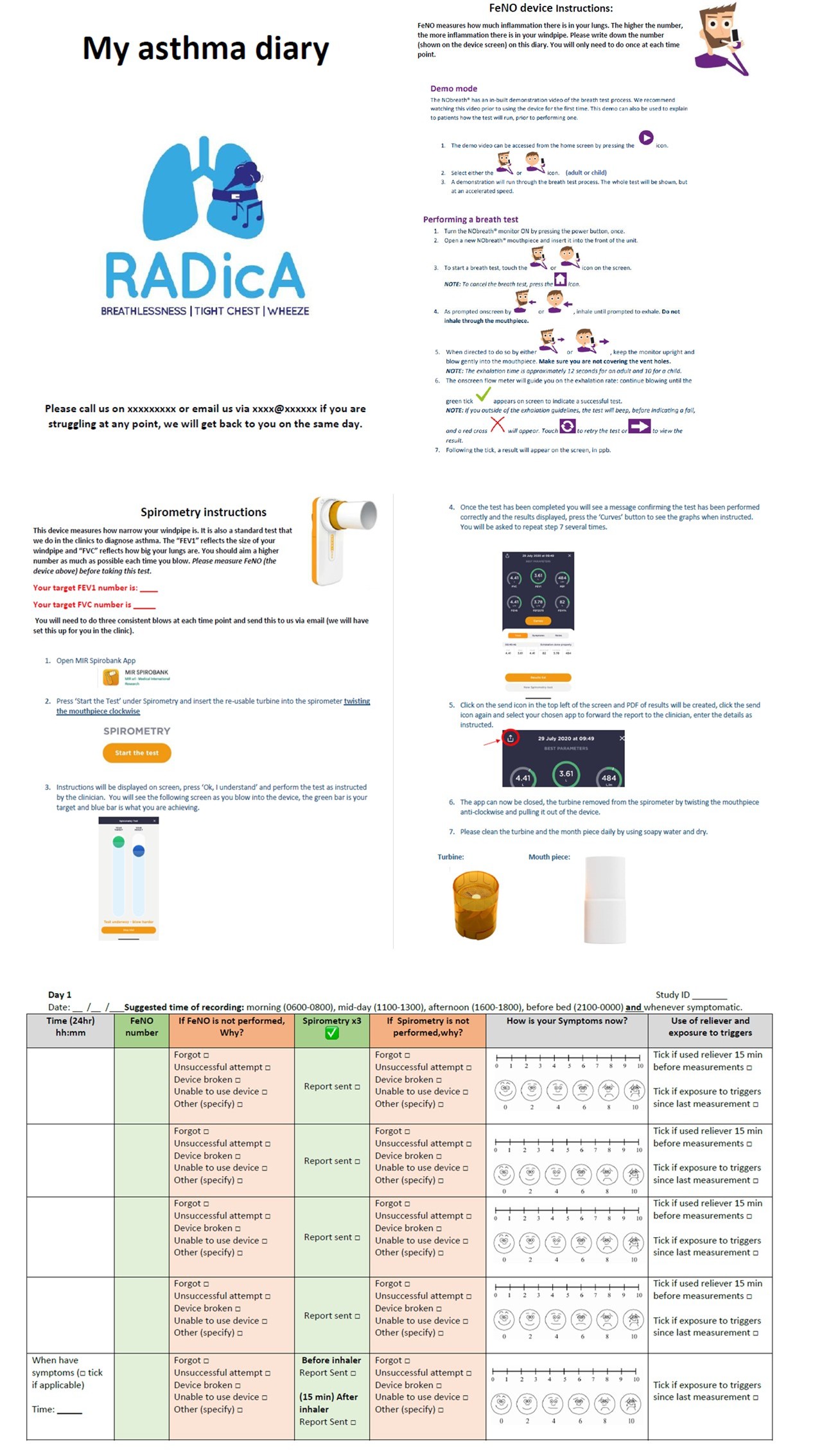

Supplement: online supplemental file 1 [file bmjopen-16-3-s001.jpg]
