# Supplementary material for: What do patients think about home-based testing for better asthma diagnosis? Insights from a qualitative study
Source: BMJ Open. 2026 Mar 23;16(3):e109347. doi: 10.1136/bmjopen-2025-109347 (PMC13034331; doi:10.1136/bmjopen-2025-109347)
Supplement: Supplementary file 2 [file bmjopen-2025-109347.reviewer_comments.pdf]

PEER REVIEW HISTORY

BMJ Open publishes all reviews undertaken for accepted manuscripts. Reviewers are asked to complete a checklist review form and are provided with free text boxes to elaborate on their assessment. These free text comments are reproduced below.

ARTICLE DETAILS

Title (Provisional)

What do patients think about home-based testing for better asthma diagnosis? - Insights from a qualitative study

Authors

Khatoon, Binish; Smith, Joanna; Fowler, Stephen; Simpson, Angela; Murray, Clare; Wang, Ran

---

VERSION 1 - REVIEW

**Reviewer**	**1**
**Name**	**Pappalardo, Andrea**
**Affiliation**	**University of Illinois System**
**Date**	**04-Oct-2025**
**COI**	**None**

Great use of qualitative inquiry here. I appreciate this.

Some areas to address:

Introduction:

First paragraph seems abrupt, I think just saying asthma is common and symptoms variable, which leads to potential mis diagnosis. But then be a bit more obvious about how this misdiagnosis leads to unnecessary risk more specifically. That will set up your much more detailed explanation in the 2nd paragraph better and our readers can clearly say yes this is unacceptable from the get-go.

The 2nd paragraph is too long and tries to incorporate the two reasons the diagnosis is often mis-categorized, I would be obvious in the first sentence asthma is mis-diagnosed due to the clinical presentation and the classic approach to clinical and diagnostic assessment. Right now it seems to go back and forth, and it may be worth breaking up these paragraphs for readability. The concepts are very good though. Wherever you can reduce some of the text here, it may be a good idea.

As you go into your next paragragh, your focus is on test timing. It is accurate, but you are narrowing yourself too much. The idea is that since the current set-up isn't sufficient to

accurately diagnosis, we must seek an innovative way to figure this all out. We have the techology, but we have no idea what adult patients would think about this given their newness to this process. The other issue with this paragraph is that your introducing the home-based testing which I think if you flipped this concept would make your point more obvious.

Methods:

--Although you state how you created this interview guide, you don't explain what is actually in it. That is necessary for this paper even if you put the guide in supplemental files. This helps orient the readers as to what you were asking specifically or at least topically to address your research question.

How was the codebook created? This information is missing. How did you ensure accuracy and concordance with only one coder, this is a major weakness. This is a group process, how many people where helping them theme? How many meetings were there to ensure consensus? This is a weakness that must be resolved.

What framework was used to create themes and to code?

I do not understand how you divided these by exploratory account, etc within this sub-section of description of the coding to theming. I think because it lacks a framework that guided this process and clear explanation by when the whole group was involved.

Patient and Public Involvement Section: This is very good you did this and I applaud you, but how is this relevant to the qualitative portion of this part of the study? It seems like it could be a one sentence addition to the larger study you mention above and use this space otherwise to be much better at desribing your qualitative methods more rigorously. The goal is that I could easily repeat this study, and I cannot say that I can with the information provided. It may be good if BK is the one who writes this section so that they can ensure it is detailed enough to be recreated.

Results:

Here is where you shine, great descriptions of the findings, but I would also like to see how many people within the n of 15 are bringing up these. This will help me understand the code presence and weight of whats being said by one or two people vs many. That helps quantify a bit of the strength of what you're finding. I would do this throughout.

Point of curiosity: why did you do both devices? Seems like that may be confusing to them. I think the spirometer would be much more beneficial to have at home than the FENO. I wonder if you could explain this reasoning a bit in the methods. This is confusing. Also, what was the training for the devices like? May also want to bring that up in the methods. as I read the quotes, this is what comes to mind.

Discussion is sufficient, but I think you revealed a huge issue in the results that was not addressed here. The feeling that asthma is not a big deal and not life threatening and how

these types of interventions may not be taken seriously until we can better communicate the risks of "mild" asthma. I would like to see that also weaved in there.

**Reviewer**	**2**
**Name**	**Middleton, Peter**
**Affiliation**	**The University of Sydney, Respiratory & Sleep Medicine**
**Date**	**15-Nov-2025**
**COI**	**None**

The authors report a small study of 15 interviews to assess the patient perception of home – based spirometry and FeNO testing for a possible diagnosis of asthma. This study provides information concerning the acceptability of this approach in this small group of individuals and builds on their previous work about home based monitoring of people known to have asthma.

The original group of 51 patients is not explained how this relates to the 26 who were invited and 16 agreed, with 15 transcripts available. It is likely that those who completed interviews were a sub-selected group and may not reflect all the opinions of the people, especially those who did not perform testing. This limits any conclusion about barriers to participation. Indeed, the authors have recently shown (ref 20) that > 40% of people with asthma do not do spirometry at home – even when they have a definite diagnosis. Will this diagnostic group be more or less engaged?

Questions

1. Could the authors add the breakdown of 51 to 26 to 15 so that the reader can follow the selection process? The authors state that “all patients used home spirometry and FeNO devices during their diagnostic processes”. Could the authors interview some of the people referred to the system who did NOT perform regular testing to actually work out the true barriers? This would strengthen the qualitative approach.

2. Given the small cohort, could the authors consider using a comparator group who just undertook peak flow recordings? The obvious advantage of peak flow is the speed and simplicity of the device compared with more arduous testing with a spirometer according to ATS/ERS guidelines. Alternatively what was the comparison with the asthma group – same themes or different?

Minor points –

3. The reviewer could not find anything in the NICE guidelines (ref 8) stating that > 60% of individuals with suspected asthma will require bronchial challenge testing. Given the algorithm starts with measuring eosinophils, FeNO, spirometry before and after

bronchodilator and Peak flow variability, the reviewer finds it difficult to follow this claim of >60% require challenge testing.

4. Similarly, with (ref 18) Simpson et al, the reviewer could not find any discussion of the sensitivity of peak flow recordings as claimed in the manuscript. The reference actually compares different guidelines rather than individual tests. Could the authors please discuss?

5. In the discussion, the authors claim to highlight the key “motivators” – isn’t that the clinical team undertaking the research? Whilst the interviews have picked up some of the barriers expressed by this sub-selected group, the interviewers have not really looked at “enablers” (sic enables) and barriers in the cohort, especially in those people who did not perform the testing. Please add this as a limitation of the study.

VERSION 1 - AUTHOR RESPONSE

Reviewer: 1

Dr. Andrea Pappalardo, University of Illinois System

Great use of qualitative inquiry here. I appreciate this.

Some areas to address:
Introduction:
First paragraph seems abrupt, I think just saying asthma is common and symptoms variable, which leads to potential mis diagnosis. But then be a bit more obvious about how this misdiagnosis leads to unnecessary risk more specifically. That will set up your much more detailed explanation in the 2nd paragraph better and our readers can clearly say yes this is unacceptable from the get-go.
The 2nd paragraph is too long and tries to incorporate the two reasons the diagnosis is often mis-categorized, I would be obvious in the first sentence asthma is mis-diagnosed due to the clinical presentation and the classic approach to clinical and diagnostic assessment. Right now it seems to go back and forth, and it may be worth breaking up these paragraphs for readability. The concepts are very good though. Wherever you can reduce some of the text here, it may be a good idea.

As you go into your next paragragh, your focus is on test timing. It is accurate, but you are narrowing yourself too much. The idea is that since the current set-up isn't sufficient to accurately diagnosis, we must seek an innovative way to figure this all out. We have the techology, but we have no idea what adult patients would think about this given their newness to this process. The other issue with this paragraph is that your introducing the home-based testing which I think if you flipped this concept would make your point more obvious.

Response: We thank you for your careful review and helpful comments. In response to your comments, we have streamlined the introduction section as suggested.

Methods:
--Although you state how you created this interview guide, you don't explain what is actually in it. That is necessary for this paper even if you put the guide in supplemental files. This helps orient the readers as to what you were asking specifically or at least topically to address your research question.

Response*:* Thank you very much for your suggestion. We have now moved the interview topic guide questions from the online supplementary file into the main manuscript (Table 1).

How was the codebook created? This information is missing. How did you ensure accuracy and concordance with only one coder, this is a major weakness. This is a group process, how many people where helping them theme? How many meetings were there to ensure consensus? This is a weakness that must be resolved. What framework was used to create themes and to code? *I do not understand how you divided these by exploratory account, etc within this sub-section of description of the coding to theming. I think because it lacks a framework that guided this process and clear explanation by when the whole group was involved.*

Response : Thank you very much for your constructive comment. In response to your comments, we have now clarified the reporting of our qualitative data analysis. We have also clarified the analytic framework and coding procedure (lines 144-282).

In the manuscript we have clarified that the Framework Analysis approach guided data analysis. We have expanded the data analysis sub-section to provide the description of the analytic process; this includes how coding progressed from initial coding to refined sub-themes and final themes.

In response to comment regarding the codebook, we have added detail on how the initial codebook was developed inductively during early coding and then how codes were defined and refined iteratively, and how the changing codebook was documented and reviewed throughout the analytic process.

While initial coding was undertaken by one researcher (BK), we have now clarified that analytic rigour was enhanced through regular consultation with an independent qualitative research adviser (JS) and repeated discussions with the wider multidisciplinary research team at key stages of analysis through regular meetings. These meetings were used to review coded extracts, challenge interpretations, resolve disagreements, and reach

consensus on theme development. This process has now been described in the methods section.

We hope we have addressed all your comments

Patient and Public Involvement Section: This is very good you did this and I applaud you, but how is this relevant to the qualitative portion of this part of the study? It seems like it could be a one sentence addition to the larger study you mention above and use this space otherwise to be much better at desribing your qualitative methods more rigorously. The goal is that I could easily repeat this study, and I cannot say that I can with the information provided. It may be good if BK is the one who writes this section so that they can ensure it is detailed enough to be recreated.

Response: Thank you for your suggestions. We have now added additional details in method section (see response to the above comments) and streamlined this section to focus specifically on PPIE contributions to delivering the qualitative study (lines 285-291).

Results:
Here is where you shine, great descriptions of the findings, but I would also like to see how many people within the n of 15 are bringing up these. This will help me understand the code presence and weight of whats being said by one or two people vs many. That helps quantify a bit of the strength of what you're finding. I would do this throughout.

Response: Thank you for your constructive comment. We have now added the number of people within the n of 15 who were commenting/bringing up these discussions throughout the results section.

Point of curiosity: why did you do both devices? Seems like that may be confusing to them. I think the spirometer would be much more beneficial to have at home than the FENO. I wonder if you could explain this reasoning a bit in the methods. This is confusing. Also, what was the training for the devices like? May also want to bring that up in the methods. as I read the quotes, this is what comes to mind.

Response: Thank you. We evaluated both domiciliary FeNO and spirometry in patients because each shows clinically relevant within-person variability in patients with asthma (day-to-day, including during symptoms, and diurnal), meaning single clinic measurements may contribute to misclassification (ref # 6-9). In response, we have added a sentence to explain this (lines 164-136); we have also expanded the Methods and online supplementary material (Figure E1) to describe the participant training procedures and support provided.

Discussion is sufficient, but I think you revealed a huge issue in the results that was not addressed here. The feeling that asthma is not a big deal and not life threatening and how these types of interventions may not be taken seriously until we can better communicate the risks of "mild" asthma. I would like to see that also weaved in there.

Response: Thank you and we agree this is an important point. We have now added this in the Discussion section (lines 497-503).

Reviewer: 2

Dr. Peter Middleton, The University of Sydney

The authors report a small study of 15 interviews to assess the patient perception of home – based spirometry and FeNO testing for a possible diagnosis of asthma. This study provides information concerning the acceptability of this approach in this small group of individuals and builds on their previous work about home based monitoring of people known to have asthma.
The original group of 51 patients is not explained how this relates to the 26 who were invited and 16 agreed, with 15 transcripts available. It is likely that those who completed interviews were a sub-selected group and may not reflect all the opinions of the people, especially those who did not perform testing. This limits any conclusion about barriers to participation. Indeed, the authors have recently shown (ref 20) that > 40% of people with asthma do not do spirometry at home – even when they have a definite diagnosis. Will this diagnostic group be more or less engaged?

Response: Thank you very much for your careful review and comments. We agree that domiciliary spirometry/FeNO may not be suitable for everyone, given the technique and adherence required. But early identification of barriers and enablers with stakeholders can maximise usability; this could also potentially help with better patient selection. We also acknowledge our previous findings (Wang et al., JACI 2023, now ref 16) of low adherence during longer-term self-management monitoring. However, this is distinguished from the diagnostic context, where home testing is likely over significantly shorter period (e.g., 1-2 weeks) and may achieve higher engagement, particularly with better patient education, structured training and support. This is highlighted in lines 491-503.

Please also refer to point-to-point response below. We hope we have satisfactorily addressed all your comments.

Questions
1. Could the authors add the breakdown of 51 to 26 to 15 so that the reader can follow the selection process? The authors state that "all patients used home spirometry and FeNO

devices during their diagnostic processes". Could the authors interview some of the people referred to the system who did NOT perform regular testing to actually work out the true barriers? This would strengthen the qualitative approach.

Responses: Thank you for this helpful comment. We agree this information is very important. In response to your comments, we have now added the adherence rates for the 15 interview participants (as well as self-reported digital confidence) to Table 2. Individuals who took part in the qualitative interviews demonstrated wide range of adherence, including 3 individuals who managed minimal home spirometry testing; 1 with minimal home FeNO testing. The breakdown of the selection of participants are outlined in lines 294-296.

2. Given the small cohort, could the authors consider using a comparator group who just undertook peak flow recordings? The obvious advantage of peak flow is the speed and simplicity of the device compared with more arduous testing with a spirometer according to ATS/ERS guidelines. Alternatively what was the comparison with the asthma group – same themes or different?

Response: Thank you very much for this helpful suggestion. Our aim was to explore innovation-specific barriers and enablers to domiciliary spirometry and FeNO in people with suspected asthma, rather than to undertake a comparator evaluation; we therefore did not interview a PEF only group. We also note that, while PEF is simple and widely available, it is less sensitive than FEV_1 for detecting airflow obstruction, particularly in smaller airways, and its diagnostic sensitivity is consistently limited (highlighted in Introduction section, lines 125–132). This underpins the need for such innovation. We did not compare themes between individuals with asthma versus not asthma because diagnostic testing was undertaken prior to diagnostic confirmation in the clinical study; this is also true in routine clinical settings.

Minor points –
3. The reviewer could not find anything in the NICE guidelines (ref 8) stating that > 60% of individuals with suspected asthma will require bronchial challenge testing. Given the algorithm starts with measuring eosinophils, FeNO, spirometry before and after bronchodilator and Peak flow variability, the reviewer finds it difficult to follow this claim of >60% require challenge testing.

Response: Thank you for highlighting this. This data was published on NICE 2024 cost utility analysis document, accessible through: https://www.nice.org.uk/guidance/ng245/evidence/costutility-analysis-most-costeffective-sequence-or-combination-of-tests-to-diagnose-asthma-pdf-13558289293, Table 36, page 55. However this reference is now taken out following streamlining the Introduction section.

4. Similarly, with (ref 18) Simpson et al, the reviewer could not find any discussion of the

sensitivity of peak flow recordings as claimed in the manuscript. The reference actually compares different guidelines rather than individual tests. Could the authors please discuss?

Response: Thank you very much for your careful review. This data is reported on the online supplement of this study, please refer to Table E2. NICE 2024 also synthesised all evidence showing similar sensitivity, which we have now added to the reference list. (https://www.nice.org.uk/guidance/ng245/evidence/evidence-review-c-accuracy-and-clinical-and-cost-effectiveness-of-peak-expiratory-flow-in-the-pdf-13558146736) Table 4. We have refined the wording accordingly (lines 130-2).

5. In the discussion, the authors claim to highlight the key "motivators" – isn't that the clinical team undertaking the research? Whilst the interviews have picked up some of the barriers expressed by this sub-selected group, the interviewers have not really looked at "enablers" (sic enables) and barriers in the cohort, especially in those people who did not perform the testing. Please add this as a limitation of the study.

Response: Thank you for your comment. We agree that our findings only reflect the perspectives of a sub-selected interview sample rather than the full study cohort. In response to your comments, we have included this as a limitation of the study (lines 515-520); this is also highlighted in the strengths and limitation bullet points. We have also added discussion of the limitation that our sample did not include participants with self-reported low digital confidence, and that future studies should specifically target this population to explore their barriers and enablers to ensure an equitable delivery of digital healthcare (Lines 521-26).

We have also clarified that "motivators" refers to patients' perceived motivators/values to undertake home testing. The enablers were explored by eliciting participants' views and suggestions for future improvements to support an effective home-testing strategy (Table 1; topic guide).

VERSION 2 - REVIEW

**Reviewer**	**1**
**Name**	**Pappalardo, Andrea**
**Affiliation**	**University of Illinois System**
**Date**	**04-Feb-2026**
**COI**	

This is an appropriately timed qualitative study assessing perceptions of people with asthma on using home-testing techniques in asthma to assist diagnosis.

Abstract:

Representative.

Would recommend adding "the" before framework appraoch to be clear you are using a recognized QDA approach.

Introduction:

First paragraph mentions asthma misdiagnosis, this implies both over and under diagnosis, but you focus on 1 in 3 being over-diagnosed. I would mention both. You don't want to miss the diagnosis and not treat it (consequences) and you don't want to treat and give meds when its not asthma (consequences).

Asthma is a clinical diagnosis supported by lung function testing, I would also point this out more clearly. However, given the variabilty its hard to figure out if common symptoms are asthma or not without supportive testing, like spirometry or FeNO. Best timing for this is with active symptoms and the AM, but this is not always feasible. I would reduce the PEF discussion to, PEF may be more accessible, but there are significant limitations that make it more of a tool for ongoing management and not for diagnosis.

2nd paragraph: describe the two diagnostic tools for asthma you have here, how they are traditionally used, how tech has advanced home use, and how the UK initiative supports this. However, there aren't many studies exploring how adults with asthma feel about all of this and if this is even a viable option for folks.

Last "paragraph" I would frame the gap you are trying to fix better, the WHY you are looking into this and how you feel this will impact next steps in the deployment of such tech.

Methods:

Participant Recruitment section is confusing and goes from parent study to spiro applications and downloading software to participants, sub-headings and going through each step of the study methodically would be helpful to follow better here.

Why were participants asked to do FeNO four times a day and Spiro 2 times a day and prn? What was the reasoning for this intensity of home-based testing? This could limit feasibilty and may not represent real-world perceptions given that this approach wouldn't be used in clinically.

Throughout methods there are not enough citations related to the techniques and COREG you are using.

What was the reasoning for only having one main coder and thematic analysis team member only checking in with one other person? Why werent asthma people included to understand

that nuance? How many people were in the team meetings? Who were these individuals/intersectionality/expertise? Was the PAG member helping this analysis?

How were results interpreted with the help of the PAG? How were results shared?

Results:

What does with a diverse background mean? Diversity as to which demographic(s)? Do you have any collected information on these participants you could share to assess whether thematic analysis differed by demographics? Be sure to reference Table 2 here.

I would like a figure instead of the asthma diary of the main takeaways related to home-based spiro and FeNO.

Results were otherwise excellently described and supported by quotations that demonstrated the points.

One area I would encourage to consider solutions toward was the peceptions of overwhelm and stigmatization, how could those barriers be overcome? Any solutions they recommended for these that could be highlighted along with the technical issues and need for in-person and leaflets?

Discussion:

I would re-write the first paragraph with this sentence leading "We found that home-based..." That this means that this is a feasible and acceptable option and that this aligns with the UK iniative. I like to lead with the main takeaway and then apply it to the larger impact, as it flows better since we just read results.

Otherwise, what discussion lacks is taking your comparison further, what is known about perceptions about home devices that may be used in other settings/ in COPD or other lung diseases? In asthma? How do your results compare or contrast compared to these? I find a lot of influence on your own study and past findings in others, but not this larger impact lens and comparison to other studies on this type of work.

**Reviewer**	**2**
**Name**	**Middleton, Peter**
**Affiliation**	**The University of Sydney, Respiratory & Sleep Medicine**
**Date**	**19-Jan-2026**
**COI**	

---

the reviewer thanks the authors for their adjustments and hope that his clarifies the outcomes..

No further comments

VERSION 2 - AUTHOR RESPONSE

Reviewer: 1

Dr. Andrea Pappalardo, University of Illinois System

Comments to the Author:

-This is an appropriately timed qualitative study assessing perceptions of people with asthma on using home-testing techniques in asthma to assist diagnosis.

Response:
Thank you very much for your thorough review of our manuscript and your helpful comments. In response to your comments, we have made additional changes - please see point-to-point response. We hope we have improved the clarity of our manuscript and have addressed all your comments satisfactorily.

The line numbers refer to corresponding lines in the clean version of the uploaded document.

-Abstract:

Representative.

Would recommend adding "the" before framework approach to be clear you are using a recognized QDA approach.

Response: Added.

-Introduction:
First paragraph mentions asthma misdiagnosis, this implies both over and under diagnosis, but you focus on 1 in 3 being over-diagnosed. I would mention both. You don't want to miss the diagnosis and not treat it (consequences) and you don't want to treat and give meds when its not asthma (consequences).

Response: Thank you for your suggestion. We have now added this information and corresponding reference (lines 99-101, ref #5).

Asthma is a clinical diagnosis supported by lung function testing, I would also point this out more clearly. However, given the variabilty its hard to figure out if common symptoms are asthma or not without supportive testing, like spirometry or FeNO. Best timing for this is with active symptoms and the AM, but this is not always feasible. I would reduce the PEF discussion to, PEF may be more accessible, but there are significant limitations that make it more of a tool for ongoing management and not for diagnosis.

Response: Thank you for your suggestions. We have restructured the section in response to your comments and simplified the paragraph about PEF (lines #102-115). We did not mention about its use limited in ongoing management as this is also no longer recommended as a routine monitoring strategy in BTS/NICE/SIGN 2024.

-2nd paragraph: describe the two diagnostic tools for asthma you have here, how they are traditionally used, how tech has advanced home use, and how the UK initiative supports this. However, there aren't many studies exploring how adults with asthma feel about all of this and if this is even a viable option for folks.

Last "paragraph" I would frame the gap you are trying to fix better, the WHY you are looking into this and how you feel this will impact next steps in the deployment of such tech.

Response: Thank you for your comments. We have now structured these paragraphs in response to your comments (lines# 116-129)

Methods:
Participant Recruitment section is confusing and goes from parent study to spiro applications and downloading software to participants, sub-headings and going through each step of the study methodically would be helpful to follow better here.

Why were participants asked to do FeNO four times a day and Spiro 2 times a day and prn? What was the reasoning for this intensity of home-based testing? This could limit feasibilty and may not represent real-world perceptions given that this approach wouldn't be used in clinically.

Response: Thank you for your suggestions. The method section is now structured under sub-headings:"Participant recruitment and clinical test procedures" describes recruitment procedures and test procedures; "Qualitative interviews" encompasses interview procedures.

Both home spirometry and FeNO are instructed to be performed 4 times a day for the first week [as stated in the method section: "Participants were asked to perform FeNO and spirometry tests 4 times a day, e.g. 4-5 hourly (±1hr) when awake (e.g. 0600-0800, 1100-1300, 1600-1800, 2100-2400hr) and whenever symptomatic (before salbutamol use) for one week."]. For the second week, we asked participants to do twice daily and whenever symptomatic. This schedule was designed to mirror the established two-week peak expiratory flow (PEF) protocol used in routine asthma diagnosis to assess diurnal variability. As PEF monitoring over two weeks remains a recognised diagnostic parameter, we sought to evaluate whether digital spirometry and FeNO could feasibly capture equivalent longitudinal variability within a similar timeframe. The four-times-daily schedule in the first week is to

enable characterisation of diurnal patterns in these test parameters and identification of potentially optimal measurement time points for future refinement. In response to your comments, we have included this information in the manuscript (lines #160-166).

We agree that testing frequency influences feasibility and participant burden. However, this must be balanced against clinical utility, as too few data points may fail to capture meaningful variability and therefore limit diagnostic value. A key aim of this study was to explore perceived testing burden and acceptability at this intensity, as reported in the Results, to help inform optimisation of frequency and duration in future study design.

-Throughout methods there are not enough citations related to the techniques and COREG you are using.

What was the reasoning for only having one main coder and thematic analysis team member only checking in with one other person? Why werent asthma people included to understand that nuance? How many people were in the team meetings? Who were these individuals/intersectionality/expertise? Was the PAG member helping this analysis?

How were results interpreted with the help of the PAG? How were results shared?

Response: Thank you for your comments. We have now clarified coding methods and rationale in the manuscript (lines #240-245). We have also now added a dedicated subheading of "strengths and limitation" in Discussion section to acknowledge some of the limitation raised in this review. Although PAG members were involved in study design and delivery, they were not involved in data interpretation; this is acknowledge in the limitation section (lines: #494-496).

-Results:
What does with a diverse background mean? Diversity as to which demographic(s)? Do you have any collected information on these participants you could share to assess whether thematic analysis differed by demographics? Be sure to reference Table 2 here.

Response: Thank you for your comments. We have reworded this sentence in the Result section to "Data collected from 15 participants with varying level of demographic background, digital confidence and differing adherence with home testing were included in the analysis (Table 2)." Diversity of sampling is further discussed in the strengths and limitations in the detail (lines 483-494). Our study was not powered to conduct formal

subgroup comparisons. We have now also included this in the limitation section of Discussion (line 495).

-I would like a figure instead of the asthma diary of the main takeaways related to home-based spiro and FeNO.

Response: Thank you for this helpful suggestion. The diary was included to illustrate the practical aspects of home testing, including the procedures, written instructions, support provided and recording requirements. These elements directly reflect participant burden and training needs, and we felt that these are important for contextualising the themes identified in our qualitative analysis. For this reason, we felt it was valuable to retain the diary in the online supplement to provide readers with a clearer understanding of the participant experience.

The key patient-facing components relate primarily to the home spirometry and FeNO devices and their associated mouthpieces. In response to your comments, we have now added this information in the Online supplement Figure E1 legend narratively.

We would be very happy to develop an alternative figure highlighting these elements and the main takeaways regarding home-based testing. If the reviewer could clarify their preference for how this information should be presented, we would gladly adapt the materials accordingly.

- Results were otherwise excellently described and supported by quotations that demonstrated the points.

One area I would encourage to consider solutions toward was the peceptions of overwhelm and stigmatization, how could those barriers be overcome? Any solutions they recommended for these that could be highlighted along with the technical issues and need for in-person and leaflets?

Response: Thank you very much for your feedback. We did not further investigate solutions to overcome feelings of overwhelm or stigmatisation within the study. We have further discussed this issue the Discussion section (lines 474-476).

-Discussion:
I would re-write the first paragraph with this sentence leading "We found that home-based..." That this means that this is a feasible and acceptable option and that this aligns with the UK iniative. I like to lead with the main takeaway and then apply it to the larger impact, as it flows better since we just read results.

Otherwise, what discussion lacks is taking your comparison further, what is known about perceptions about home devices that may be used in other settings/ in COPD or other lung diseases? In asthma? How do your results compare or contrast compared to these? I find a lot of influence on your own study and past findings in others, but not this larger impact lens and comparison to other studies on this type of work.

Response: Thank you very much for your helpful comments. We have restructured the discussion section in response to your comments. We have also now highlighted the similarities and differences from previous studies of home-based devices in other chronic lung diseases and included additional references.

Thank you again for your thorough review. We hope we have addressed your comments satisfactorily.
